# Supplementary material for: Predicting clinical benefit of immunotherapy by antigenic or functional mutations affecting tumour immunogenicity
Source: Nat Commun. 2020 Feb 19;11:951. doi: 10.1038/s41467-020-14562-z (PMC7031381; doi:10.1038/s41467-020-14562-z)
Supplement: Supplementary file 1 — Supplementary Information [file 41467_2020_14562_MOESM1_ESM.pdf]

Supplementary Information

Predicting clinical benefit of immunotherapy by antigenic or functional mutations affecting tumour immunogenicity

K Kim et al.

Supplementary Figure 1

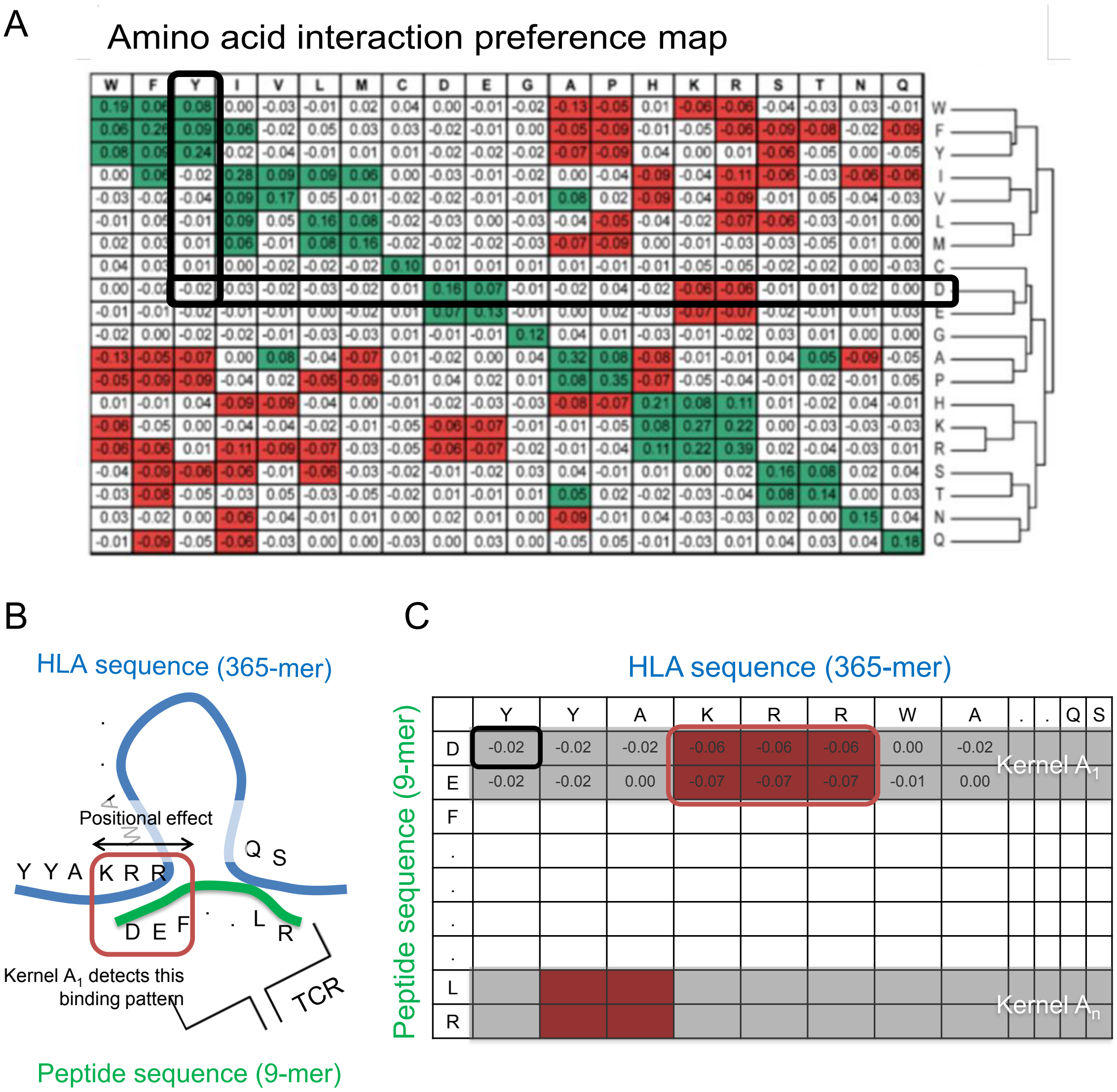

Input structure of prediction model for peptide-MHC class I binding.

(A) Amino acid interaction preference map (Vishveshwara, S. et al. Protein Sci 2010). The value of preference among 20x20 amino acid pairs in single protein was available to use as a surrogate of interacting preference in peptide-MHC class I binding. (B) Biological model for peptide-MHC class I binding. The kernel of our algorithm (convolutional neural network; CNN) detects speicific binding pattern having high interaction preference in amino acid level with positional effect. (C) An input matrix for training data. The values of the matrix were filled from the amino acid interaction map (black box in Fig A and C). A variety of kernels were used to detect binding patterns between peptide and MHC class I in amino acid level, having high interaction preferences with positional effect (red box in Fig B and C).

Supplementary Figure 2

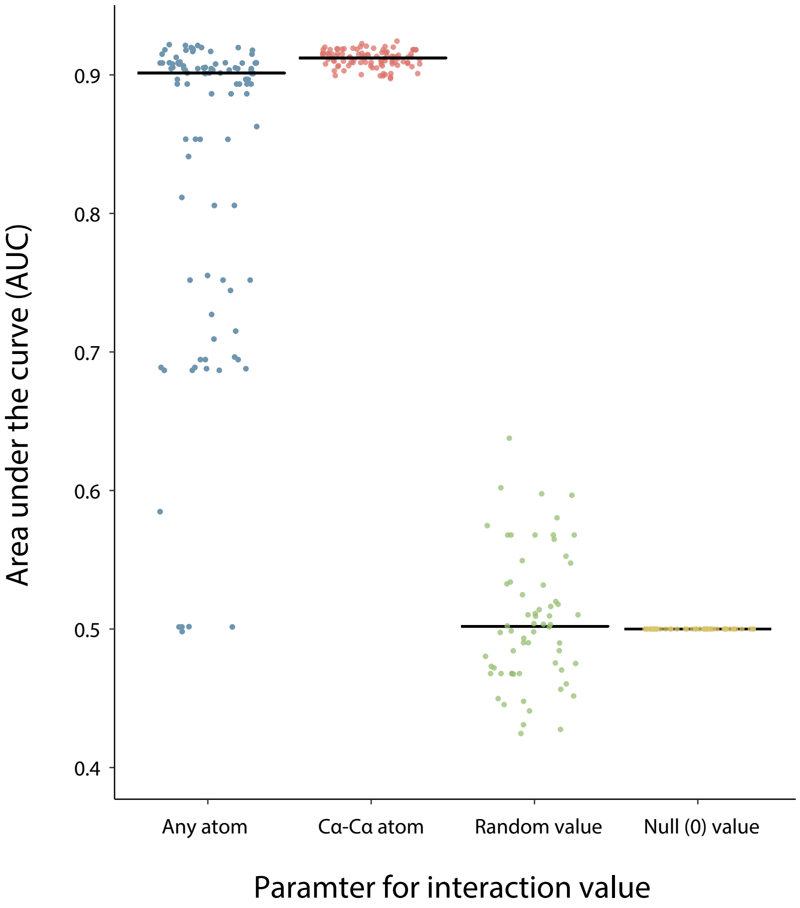

Performance of neoantigen prediction depending on the amino acid interaction map.

Amino acid interaction preferences were computed based on contacts between Cα atoms or between any atoms from native protein structures. We also prepared interaction maps consisting of randomly permuted or null values. To compare the four different input matrices, we performed CNN training with 100 hyperparameters. Each dot marks AUC in each validation process of the prediction models with 100 hyperparameters. The horizontal lines indicate the median.

Supplementary Figure 3

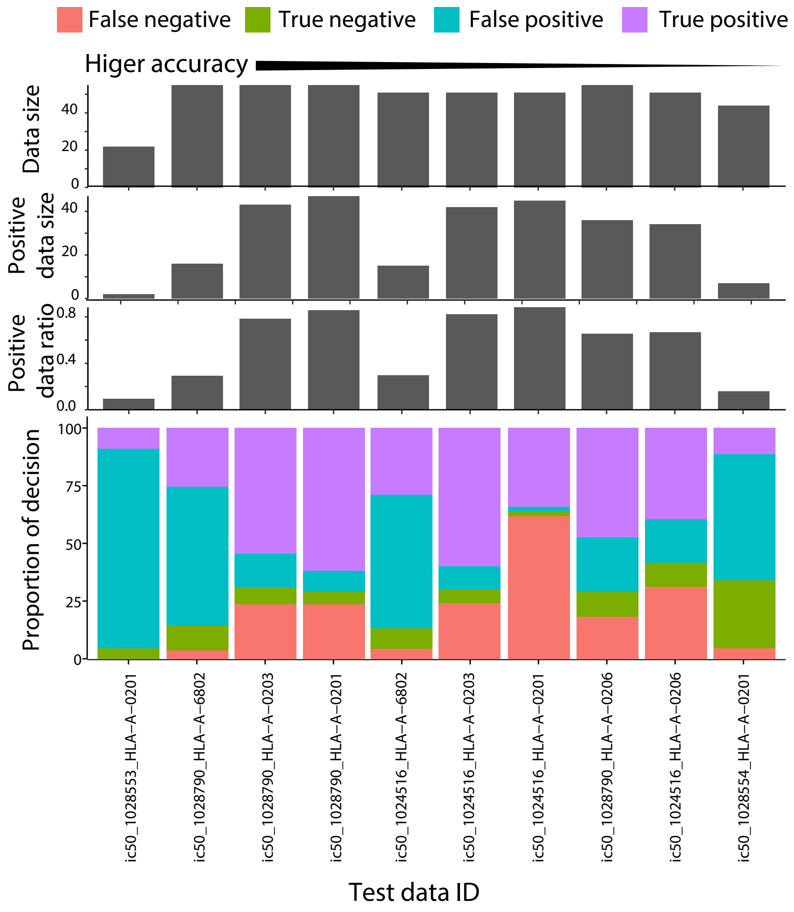

Dependence of prediction accuracy on the size of test sets.
Proportion of true positives, true negatives, false positives, and false negatives was obtained according to the size of test data. The data size is represented as the total number of data points ("Data size"), the number of binding (positive) data points ("Positive data size"), and the fraction of the binding data points ("Positive data ratio"). The test data IDs are shown at the bottom in the order of prediction accuracy. The test data is grouped by three categories of binding experiments in accordance with different length of peptide and different class of HLA.

Supplementary Figure 4

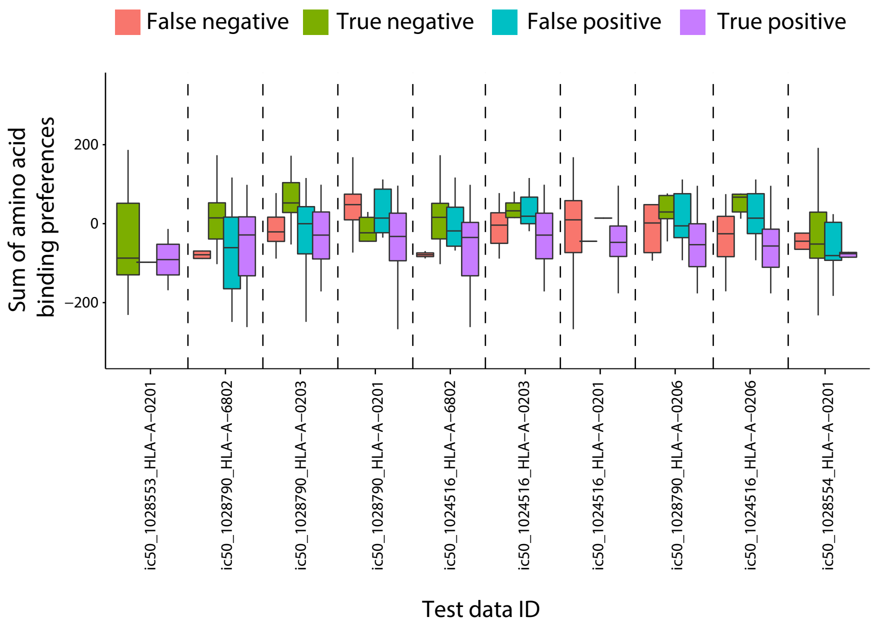

Effects of aggregate amino acid preferences on neoantigen prediction.

The sum of the amino acid preferences was calculated for the cases corresponding to true positive, true negative, false positive, and false negative. Lower values of binding energy correspond to higher preferences in binding between peptides and MHC class I proteins.

Supplementary Figure 5

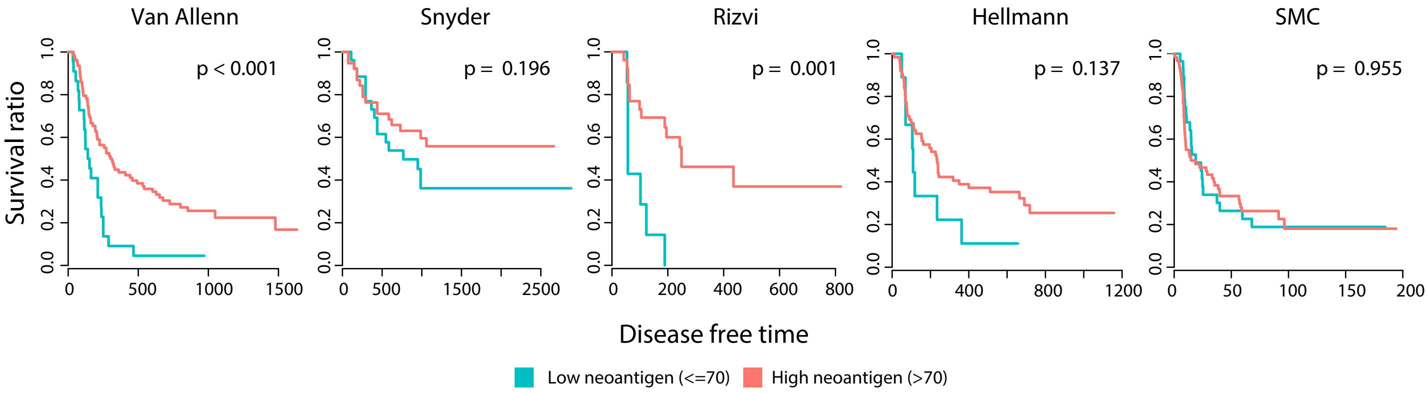

Correlation of neoantigen load calculated by NetMHCpan in melanoma and lung cancer patient survival.

Survival analysis was performed bu NetMHC for samples with high versus low neoantigen load in the two melanoma and three lung cancer clinical trials. The same threshold of NetMHC as CNN was used.

Supplementary Figure 6

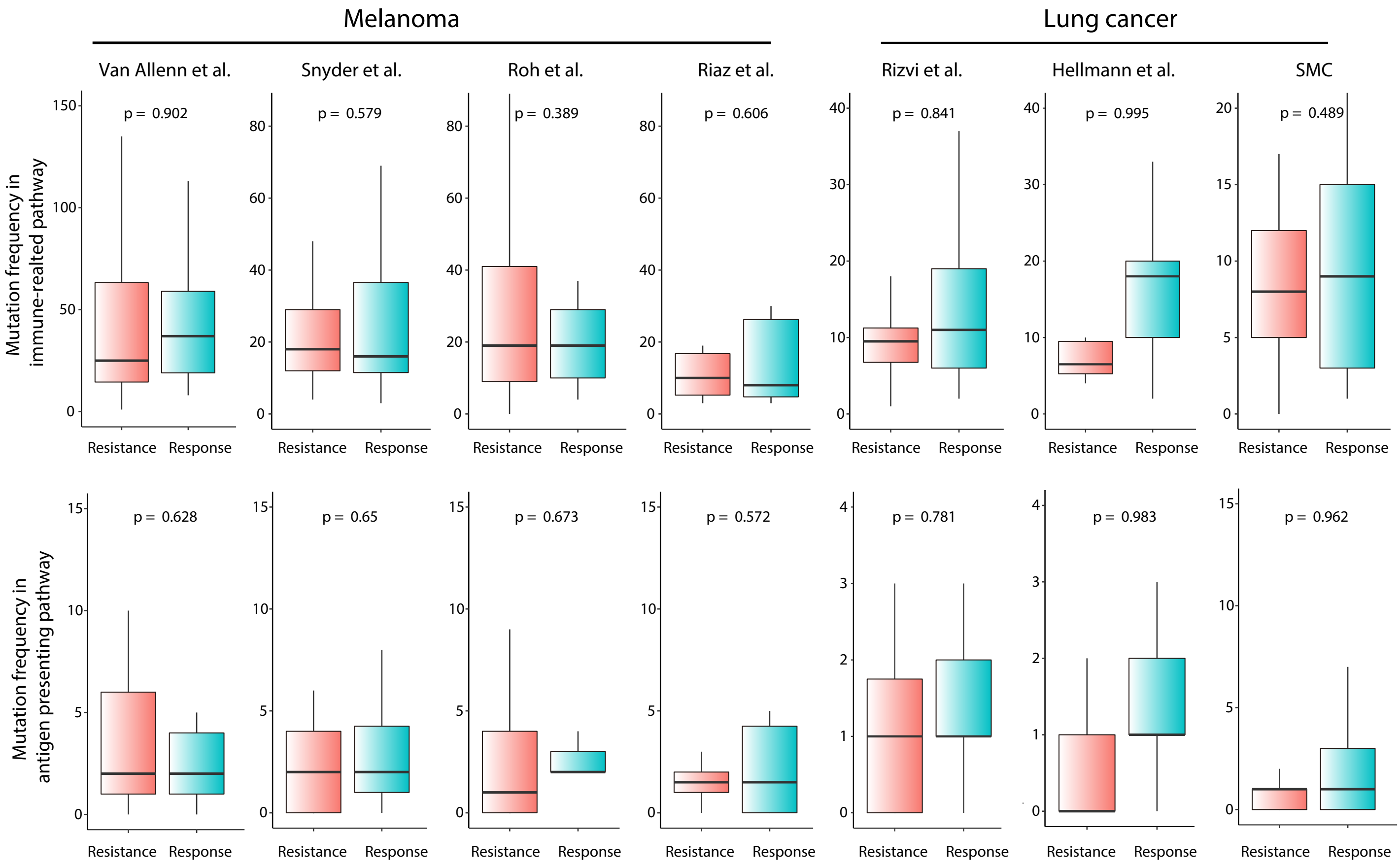

Frequency of functional mutations in immune-related genes and clinical response to immunotherapy.

Mutation load on genes in immune-response-related pathways (up) and genes in antigen presenting pathways (bottom). The melanoma and lung cancer samples with high neoantigen load were divided according to their response to immunotherapy.

Supplementary Figure 7

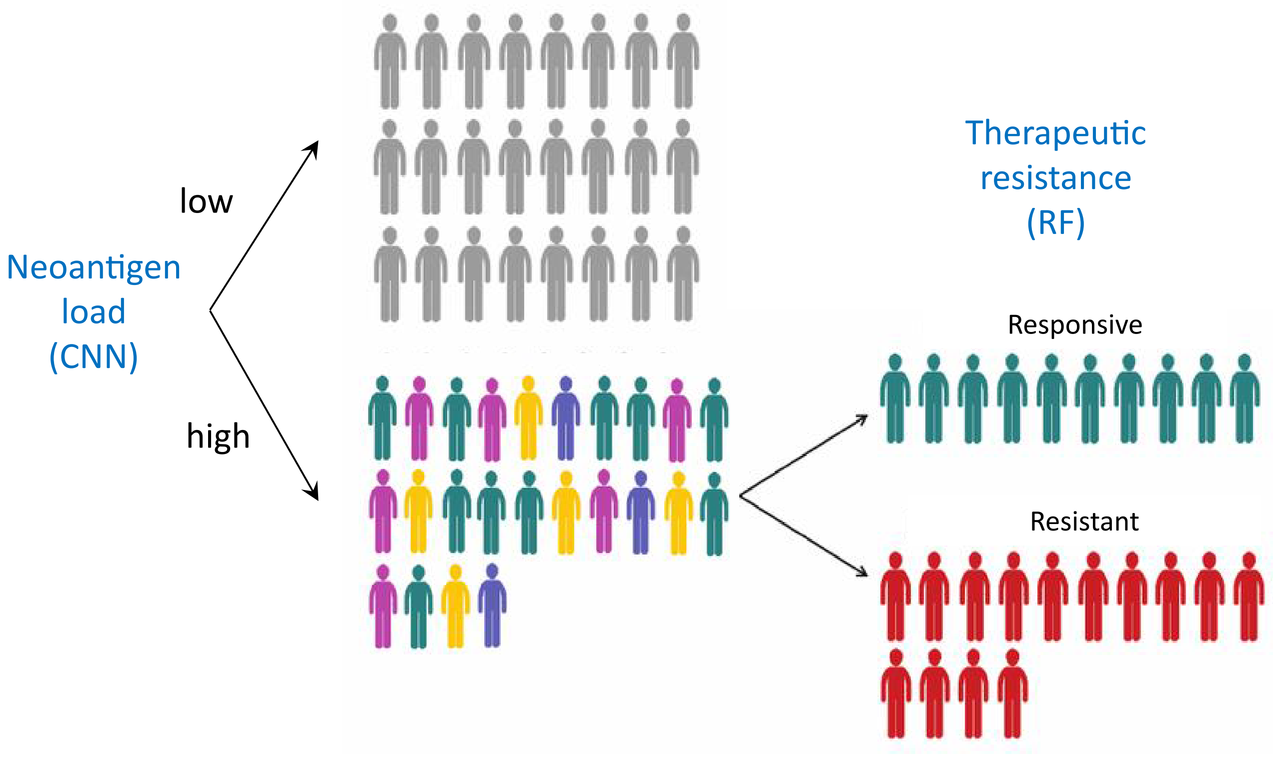

A diagram describing therapeutic resistance.

We use the term “therapeutic resistance” because patients with high neoantigen load are expected to respond to checkpoint blockade but may bear resistance because of functional mutations that promote immune evasion. The final prediction score by the RF, indicating whether the given patient would bear therapeutic resistance, was referred to as the “exomic prediction score” because exome data were used for prediction by the RF.

Supplementary Figure 8

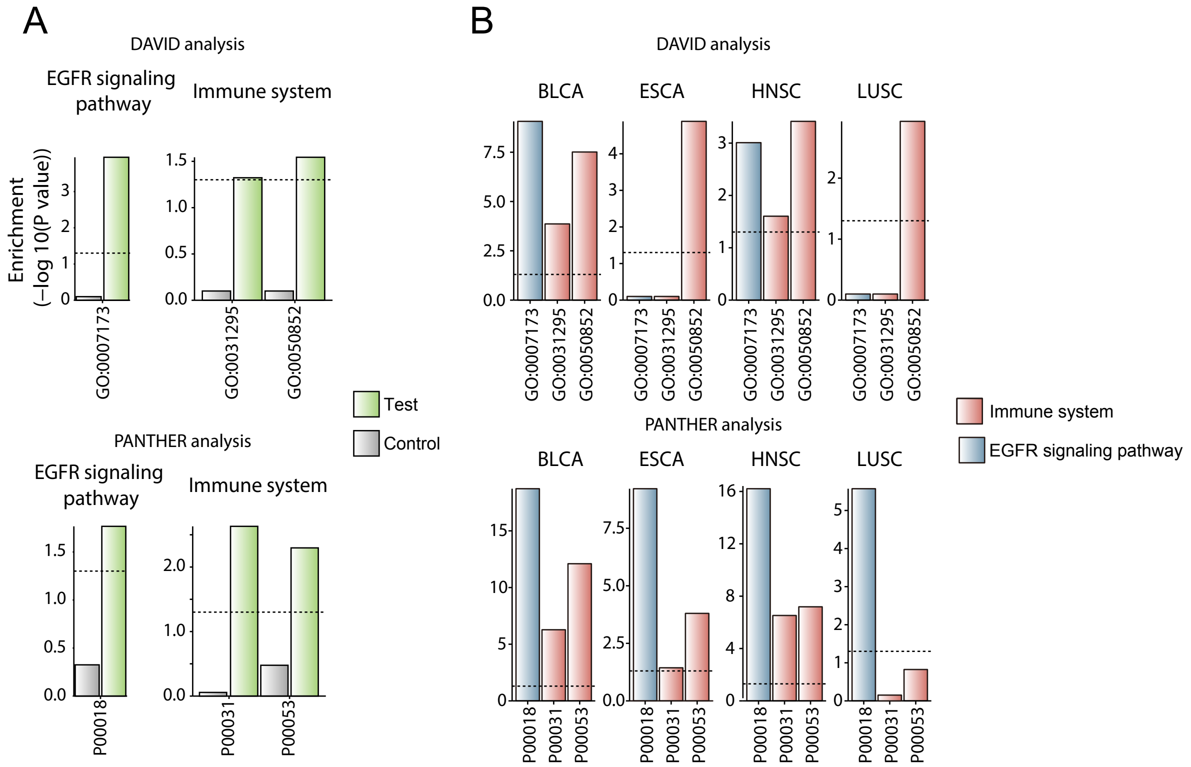

P00031 = Inflammation mediated by chemokine and cytokine signaling pathway, P00053 = T cell activation, P00018 = EGF receptor signaling pathway
GO:0007173 = epidermal growth factor receptor signaling pathway, GO:0050852 = T cell receptor signaling pathway, GO:0031295 = T cell costimulation

Functional enrichment of genes with high explanatory power.

Functional enrichment analyses for the genes with high explanatory power and their interacting partners were performed by two independent web-based tools (upper panel for DAVID and lower panel for PANTHER). We analyzed (A) the two melanoma cohorts and (B) TCGA samples of different tumor types.
