# Supplementary material for: Predicting clinical benefit of immunotherapy by antigenic or functional mutations affecting tumour immunogenicity
Source: Nat Commun. 2020 Feb 19;11:951. doi: 10.1038/s41467-020-14562-z (PMC7031381; doi:10.1038/s41467-020-14562-z)
Supplement: Supplementary file 3 — Description of Additional Supplementary Files [file 41467_2020_14562_MOESM3_ESM.pdf]

Description of Additional Supplementary Files

File Name: Supplementary Data 1
Description: Information of our lung cancer cohort

File Name: Supplementary Data 2
Description: 109 important genes and their partners for prediction in lung cancer

File Name: Supplementary Data 3
Description: 161 important genes and their partners for prediction in melanoma

File Name: Supplementary Data 4
Description: SNV/indel calling results for our cohort

File Name: Supplementary Data 5
Description: CNV calling results for our cohort

File Name: Supplementary Data 6
Description: Neoantigen calling results for our cohort
